# Supplementary material for: First-Principles Analysis of Chirality-Induced Spin Selectivity at Molecule–Metal Interfaces in Photoemission
Source: Nano Lett. 2026 Jul 10;26(28):9059–65. doi: 10.1021/acs.nanolett.6c01438 (PMC13397889; doi:10.1021/acs.nanolett.6c01438)
Supplement: Supplementary file 1 [file nl6c01438_si_001.pdf]

Supporting Information: First-Principles Analysis of Chirality-Induced Spin Selectivity at Molecule-Metal Interfaces in Photoemission

Amos Afugu, Gyanu P. Kafle, and Zhen-Fei Liu*

Department of Chemistry, Wayne State University, Detroit, Michigan 48202, USA

E-mail: zfliu@wayne.edu

Theoretical Details on Computing Photoemission

Within the three-step photoemission model and the independent-particle approximation, the first step corresponds to an optical transition from an occupied initial state to an unoccupied final state. When the final state is non-degenerate, the transition probability is given by Fermi's golden rule,

$$\mathcal{P}_{\mathrm{fi}} \propto \left|\langle\phi_{\mathrm{f}}|\mathbf{A}\cdot\mathbf{p}|\phi_{\mathrm{i}}\rangle\right|^2 \delta\left(\epsilon_{\mathrm{f}} - \epsilon_{\mathrm{i}} - h\nu\right). \tag{S1}$$

Here, we have omitted the prefactor for conciseness. The first factor is the optical transition matrix element, where ϕ_{i} and ϕ_{f} denote the (non-interacting) initial and final states, respectively. They are approximated in this work by Kohn-Sham orbitals obtained from DFT, which are two-component spinors. $\mathbf{A}$ is the vector potential of the incident light, and $\mathbf{p}$ is the momentum operator. Within the dipole approximation, the light-matter coupling reduces to $\mathbf{A}\cdot\mathbf{p} = A_0(p_x \pm \mathrm{i}p_y)$ in the velocity gauge for circularly polarized light. Here, A_0

is a constant prefactor, and "+" corresponds to right circular polarization, defined for the direction of rotation of the electric vector being clockwise when viewed against the direction of light propagation. The "−" sign corresponds to left circular polarization. For linearly polarized light, we use $\mathbf{A} \cdot \mathbf{p} = A_0 p_x$ in this work (using $\mathbf{A} \cdot \mathbf{p} = A_0 p_y$ yields very similar results numerically for the systems studied here). The delta function in Eq. (S1) enforces energy conservation during the optical excitation, with ϵ_i and ϵ_f denoting the Kohn-Sham eigenvalues of the initial and final states, respectively, and $h\nu$ the photon energy.

When the final states form a degenerate manifold, transitions from a given initial state to different final states within that manifold interfere coherently.[1,2] Within a degenerate subspace $\mathcal{D}$, we therefore construct the optically excited state as

$$|\phi_{\mathrm{i}\to\mathrm{f}'}\rangle = \sum_{j\in\mathcal{D}} p_{\mathrm{i}\to\mathrm{f}_j} |\phi_{\mathrm{f}_j}\rangle = \sum_{j\in\mathcal{D}} \left(\langle\phi_{\mathrm{f}_j}|\mathbf{A}\cdot\mathbf{p}|\phi_\mathrm{i}\rangle\right) |\phi_{\mathrm{f}_j}\rangle \,. \tag{S2}$$

Here, the transition amplitude is $p_{\mathrm{i}\to\mathrm{f}_j} = \langle\phi_{\mathrm{f}_j}|p_x \pm \mathrm{i}p_y|\phi_\mathrm{i}\rangle$ for circularly polarized light and we use $p_{\mathrm{i}\to\mathrm{f}_j} = \langle\phi_{\mathrm{f}_j}|p_x|\phi_\mathrm{i}\rangle$ for linearly polarized light. It is worth noting that when the Hamiltonian $\hat{H}$ contains nonlocal terms due to the pseudopotential – as is the case in our calculations – the physically relevant transition matrix elements that we actually compute are those of the velocity operator, $\langle\phi_\mathrm{f}|\mathbf{v}|\phi_\mathrm{i}\rangle$ with $i\hbar\mathbf{v} = \left[\mathbf{r}, \hat{H}\right]$, rather than those of the canonical momentum operator.

Projecting the optically excited spinor in Eq. (S2) onto $|S_z \uparrow\rangle$ and taking the squared norm yields the spin-resolved transition weight. To be explicit, if we define $\hat{P}_\uparrow = \hat{I}_\mathrm{spatial} \otimes |S_z \uparrow\rangle \langle S_z \uparrow|$, then $\langle\phi_{\mathrm{i}\to\mathrm{f}'}|\hat{P}_\uparrow|\phi_{\mathrm{i}\to\mathrm{f}'}\rangle = \|\langle S_z \uparrow|\phi_{\mathrm{i}\to\mathrm{f}'}\rangle\|^2$. Substituting $|\phi_{\mathrm{i}\to\mathrm{f}'}\rangle$ from Eq. (S2) into this expression shows that $\langle\phi_{\mathrm{f}_j}|\mathbf{A}\cdot\mathbf{p}|\phi_\mathrm{i}\rangle$, the transition amplitudes into states within the same final-state degenerate manifold, are summed coherently[2] (summed before squaring). In contrast, contributions from different occupied initial states and distinct final-state degenerate manifolds need to be summed at the probability level (squared first and then summed) in computing $I_{\mathbf{K}_\parallel}(|S_z \uparrow\rangle)$, leading to Eq. (2) in the main text.

Additional Results for Au (111) Unit Cell

Here, we consider $\mathbf{k}_\parallel$ points away from Γ, defined in Figure S1. These specific $\mathbf{k}_\parallel$ points are chosen because $|\mathbf{k}_\parallel| = 0.02$ Å^{-1} corresponds to an acceptance angle of approximately $\theta \sim 2.3°$ for photoelectrons with $E_{\text{kin}} \sim 1$ eV in a typical PES measurement, as estimated from $|\mathbf{k}_\parallel| = \sqrt{2E_{\text{kin}}} \sin\theta$ (in atomic units).

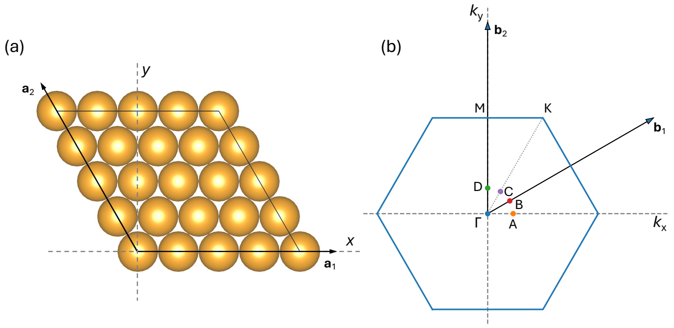

Figure S1: (a) Structure of a 4 × 4 Au (111) slab, showing real-space lattice vectors $\mathbf{a}_1$ and $\mathbf{a}_2$. (b) 2D hexagonal Brillouin zone, showing reciprocal-space lattice vectors $\mathbf{b}_1$ and $\mathbf{b}_2$ and high-symmetry $\mathbf{k}_\parallel$ points Γ, M, and K. We consider five $\mathbf{k}_\parallel$ points in the vicinity of Γ: Γ =(0, 0), A =(0.15396, −0.07698), B =(0.13333, 0), C =(0.07698, 0.07698), and D =(0, 0.13333), all expressed in fractional coordinates of $\mathbf{b}_1$ and $\mathbf{b}_2$. A, B, C, and D lie at the same radial distance from Γ, with this distance being 2/15 of $|\mathbf{b}_1|$.

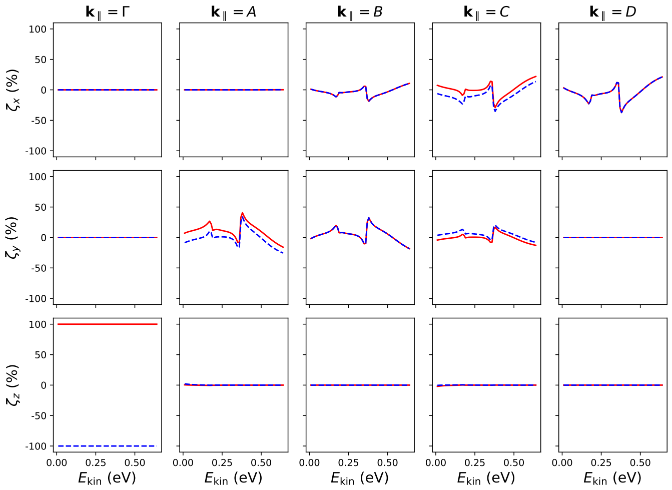

Figure S2: Spin polarization along x, y, and z directions, computed for the unit cell of nine-layer Au (111) at different $\mathbf{k}_\parallel$ points defined in Figure S1, using $h\nu = 5.83$ eV. Red (blue) lines denote ζ^+ (ζ^-), spin polarization under right (left) circularly polarized light.

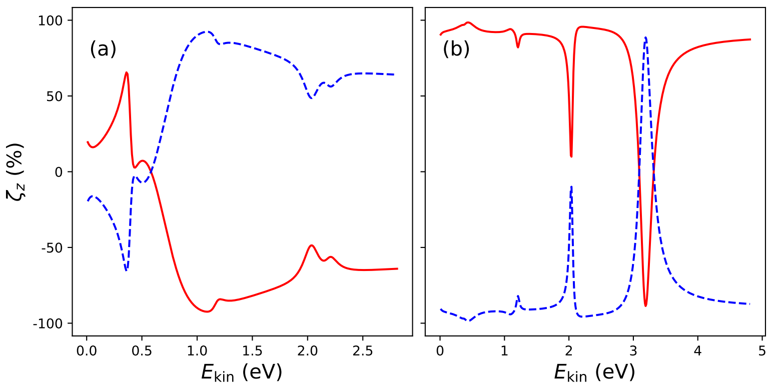

Figure S3: Spin polarization along the z direction, computed for the unit cell of nine-layer Au (111) at Γ point, for different energies of incoming photons. (a) $h\nu = 8$ eV. (b) $h\nu = 10$ eV. Red (blue) lines denote ζ^+ (ζ^-), spin polarization under right (left) circularly polarized light.

Additional Results for Helicene/Au Interfaces

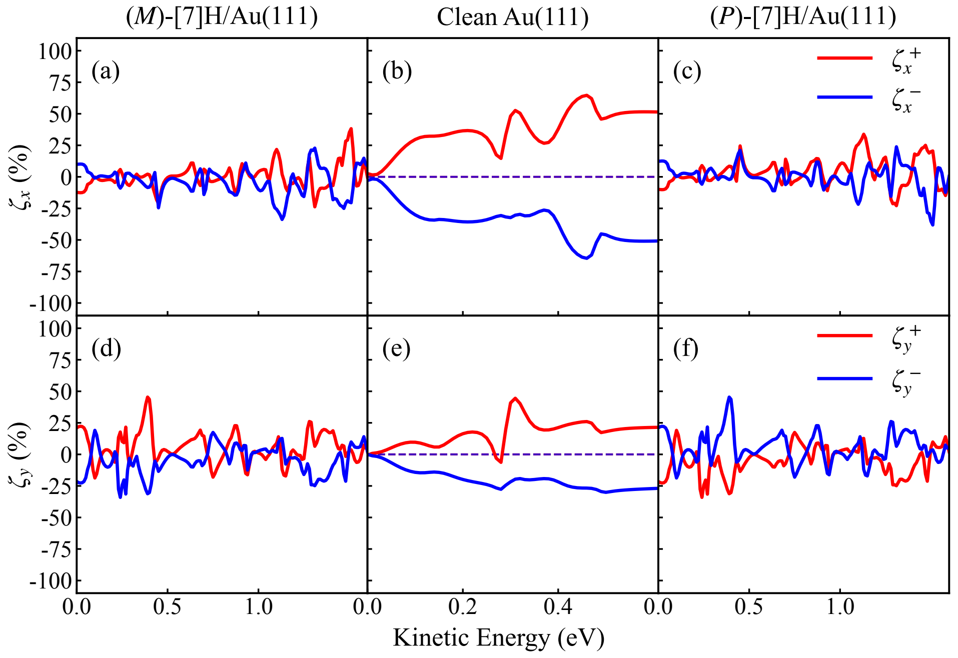

Figure S4: In-plane spin polarization along the x and y directions, evaluated at the Γ point. (a)(d) (M)-[7]H adsorbed on Au (111). (b)(e) Clean Au (111), where dashed lines are computed for a nine-layer unit cell and solid lines are computed for the Au slab with atomic coordinates adopted from the (M)-[7]H interface. (c)(f) (P)-[7]H adsorbed on Au (111). In all panels, red (blue) lines denote ζ^+ (ζ^-), spin polarization under right (left) circularly polarized light.

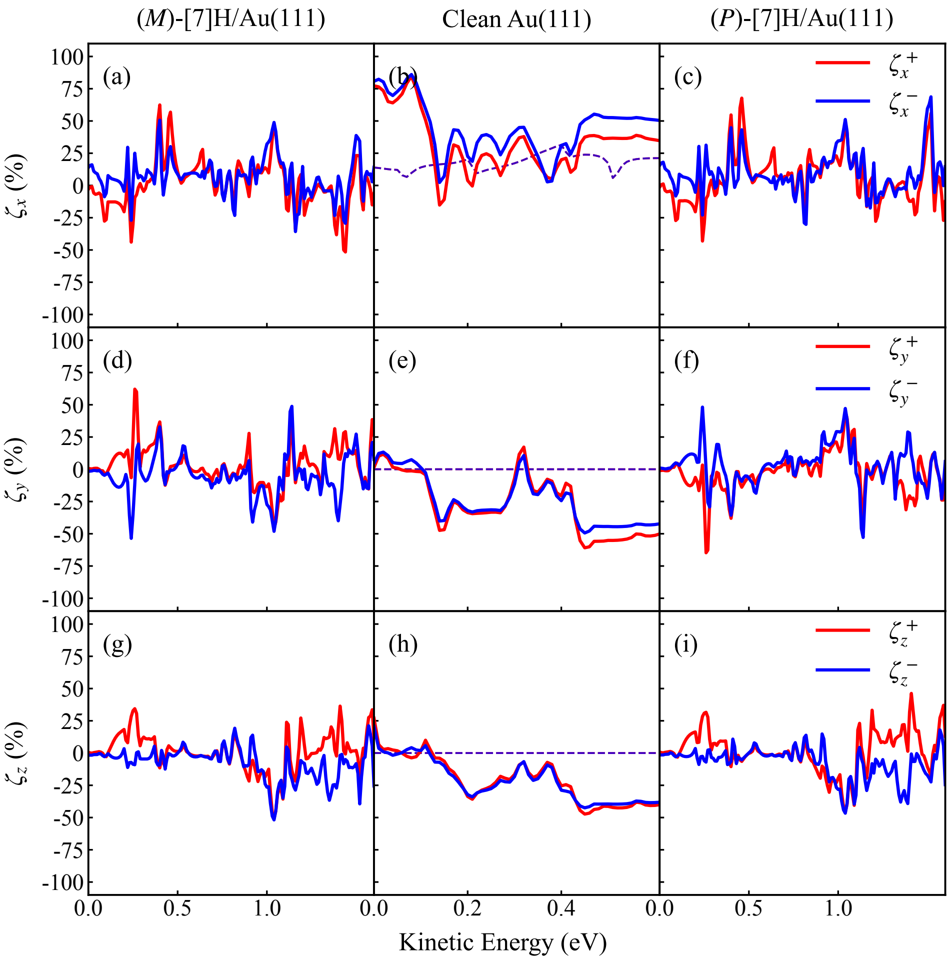

Figure S5: Spin polarizations evaluated at $\mathbf{k}_\parallel = (0, 0.03333)$, expressed in fractional coordinates of $\mathbf{b}_1$ and $\mathbf{b}_2$ in the reciprocal space. We choose this particular $\mathbf{k}_\parallel$ point again because $|\mathbf{k}_\parallel| = 0.02$ Å^{-1} (see discussions before Figure S1). (a)(d)(g) (M)-[7]H adsorbed on Au (111). (b)(e)(h) Clean Au (111), where dashed lines are computed for a nine-layer unit cell and solid lines are computed for the Au slab with atomic coordinates adopted from the (M)-[7]H interface. (c)(f)(i) (P)-[7]H adsorbed on Au (111). In all panels, red (blue) lines denote ζ^+ (ζ^-), spin polarization under right (left) circularly polarized light.

Results for Helicene/Cu(111) Interfaces

To further probe the role of substrate SOC, we investigate (M)-[7]H and (P)-[7]H adsorbed on Cu (111), a system examined experimentally in Ref. 3. The results are shown in Figure S6, presented in the same format as Figure 2 in the main text for the Au-based systems. The clean Cu (111) slab, with coordinates adopted from the (M)-[7]H/Cu interface, has a work function of 4.76 eV. Owing to the weak intrinsic SOC in Cu, it exhibits negligible

out-of-plane spin polarization. Upon adsorption of the chiral molecules, the work function is reduced to 3.60 eV, implying the maximum E_{kin} is 2.23 eV for an incident light with $h\nu =$ 5.83 eV. For the interfaces, $\zeta_z^{\pm}$ is modified to a moderate extent; however, its average over the accessible E_{kin} range remains close to zero. Moreover, the symmetry relations expressed in Eq. (4) of the main text continue to hold, as expected from the same mirror relationship between the two geometries. The ζ_z^{LP} results for the Cu-based interfaces follow the same symmetry relation as in the Au-based case but remain small, consistent with the weaker intrinsic SOC of Cu.

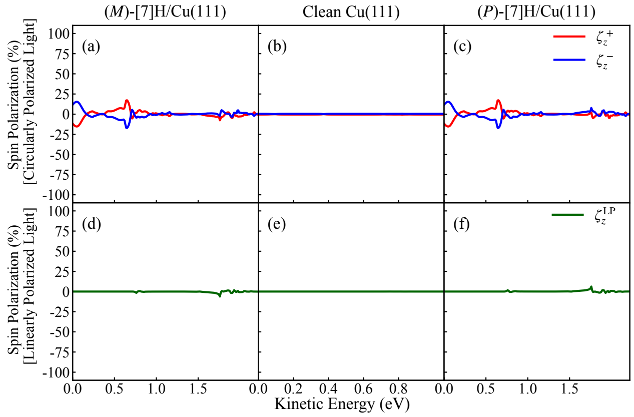

Figure S6: Spin polarization along the z direction at the Γ point. (a)(d) (M)-[7]H adsorbed on Cu (111). (b)(e) Clean Cu (111) slab with coordinates adopted from the (M)-[7]H/Cu interface. (c)(f) (P)-[7]H adsorbed on Cu (111). (a-c) Incident light is circularly polarized. Red (blue) lines denote ζ^+ (ζ^-). (d-f) Incident light is linearly polarized. This figure is analogous to Figure 2 of the main text, but for the Cu-based systems.

These results on Cu-based interfaces reinforce two points. First, they support the interpretation that the large SOC of Au is an important ingredient in generating a sizable PES spin polarization at the interface. While the current work compares Au and Cu, two materials with different SOC, it would be of future interest to quantitatively analyze the dependence of spin polarization on substrate SOC, via, e.g., varying the SOC strengths artificially and continuously. Second, even when the chiral adsorbate is present, the computed response on Cu remains much smaller than on Au, again emphasizing that the molecule cannot be viewed as the sole source of the photoemission spin polarization. Overall, our

results are consistent with the findings of Ref. 3, while also suggesting that stronger effects reported for other surfaces, such as Cu (332),[4] likely depend sensitively on the detailed interface geometry and measurement conditions.

Details of Modeling and Geometry Optimization

In Figure 1 of the main text, the Au (111) and Cu (111) substrates are modeled using nine-layer slabs, each consisting of a 4×4 lateral supercell of Au or Cu atoms. The equivalent bulk lattice constants are 4.08 Å for Au and 3.61 Å for Cu, in good agreement with experimental values. The choice of nine layers reflects the minimal slab thickness required to produce a finite spin splitting of the surface states for clean Au (111). An accurate description of the surface states is essential for the present study: in typical PES measurements probing CISS,[5] the photon energy is $h\nu = 5.83$ eV, which, given the Au (111) work function of approximately 5.3 eV,[6] restricts the accessible binding energies to within 1 eV below the Fermi level. In this narrow energy window, the spin texture of the surface states constitutes a primary source of spin polarization.[7,8] Moreover, to eliminate spurious contributions from the opposite side of the slab, the bottom metal surface is passivated with hydrogen atoms, thereby suppressing artificial surface states and ensuring that the calculated PES response originates solely from the molecule-metal interface of interest.

For geometry optimization, we model the interface systems using four layers of Au (111) or Cu (111) to reduce the computational cost. The molecular adsorbate and the top two metal layers are fully relaxed until all residual forces fall below 0.05 eV/Å, while the bottom two metal layers are kept fixed. Geometry relaxations are carried out using scalar-relativistic optimized norm-conserving Vanderbilt (ONCV) pseudopotentials,[9,10] the vdw-DF-cx functional,[11] a kinetic energy cutoff of 70 Ry, and a **k**-point mesh of $4 \times 4 \times 1$. We explicitly relax the (M)-[7]H/Au, coronene/Au, and (M)-[7]H/Cu interfaces, while the corresponding (P)-[7]H interface structures are generated by taking the mirror image of the (M)-[7]H

configurations with respect to the xz plane.

In the optimized structures, the closest carbon-to-metal distance along the z direction is 2.81 Å, 3.23 Å, and 2.34 Å for the (M)-[7]H/Au, coronene/Au, and (M)-[7]H/Cu interface, respectively. After relaxation, five additional Au (111) or Cu (111) layers are added beneath the relaxed slab in their bulk positions, yielding a total of nine metal layers in the final interface models. Additionally, the bottom metal surface is passivated with hydrogen atoms to eliminate spurious states from the opposite surface. The Au-H (Cu-H) distance is set to 1.60 Å (1.50 Å), determined from separate calculations in which a hydrogen atom is adsorbed on a ten-layer Au (111) or Cu (111) unit-cell slab and only the hydrogen coordinate is relaxed. Subsequent electronic structure calculations are performed using the Perdew-Burke-Ernzerhof (PBE) functional[12] and fully relativistic pseudopotentials, including SOC. All geometry optimizations, DFT-based electronic structure calculations, and the calculations of transition matrix elements are carried out using the Quantum ESPRESSO package.[13]

Details of G_0W_0 Calculations

To reduce the computational cost, in the G_0W_0 calculations, we employ the substrate screening approach[14] for the (M)-[7]H/Au interface. In this approach, the noninteracting Kohn-Sham polarizability of the interface is approximated as the sum of the corresponding quantities of the metal substrate and the molecular adsorbate, i.e., $\chi^0_{\text{tot}} \approx \chi^0_{\text{metal}} + \chi^0_{\text{mol}}$. The calculations of the polarizabilities, dielectric functions, and self-energies are performed using the BerkeleyGW package.[15]

For the χ^0_{metal}, we compute this quantity in a primitive unit cell of Au (111) and then fold it in the reciprocal space to a $4 \times 4 \times 1$ supercell with equivalent size as the molecule-metal interface. We use a $\mathbf{q}$-point mesh of $16 \times 16 \times 1$ and a 15 Ry dielectric cutoff, and include 375 bands in the summation. We compute the $\mathbf{q} \to 0$ limit by using a denser $\mathbf{q}$-point mesh of $32 \times 32 \times 1$ and including 120 bands.

For the χ^0_{mol}, we compute this quantity in a simulation cell that has the same xy dimensions as the interface but is much smaller along the z direction. This simulation cell is 12 Å along the z direction, 1/3 the size of the (M)-[7]H/Au interface simulation cell that includes four layers of Au (111). We use a **q**-point mesh of $4 \times 4 \times 1$ and a 15 Ry dielectric cutoff, and include 2000 bands in the summation. The $\mathbf{q} \to 0$ limit is treated using semiconductor screening, with 320 bands on a shifted **q**-mesh included in the summation. We then map this χ^0 in the real space to a corresponding quantity defined in the molecule-metal interface simulation cell, following the procedure detailed in Ref. 14.

After combining χ^0_{metal} and χ^0_{mol} in the simulation cell of the molecule-metal interface, we compute the dielectric function of the interface and then the self-energies, as in standard G_0W_0 calculations. The frequency dependence of the dielectric function is described by the Hybertsen-Louie generalized plasmon pole model.[16] We employ metallic screening, a slab Coulomb truncation scheme,[17] and a static remainder approximation[18] in the self-energy calculations, which include 6000 bands of the interface in the Green's function and use a 15 Ry dielectric cutoff.

References

(1) Wang, Y. H.; Hsieh, D.; Pilon, D.; Fu, L.; Gardner, D. R.; Lee, Y. S.; Gedik, N. Observation of a Warped Helical Spin Texture in Bi_2Se_3 from Circular Dichroism Angle-Resolved Photoemission Spectroscopy. *Phys. Rev. Lett.* **2011**, *107*, 207602.

(2) Park, C.-H.; Louie, S. G. Spin Polarization of Photoelectrons from Topological Insulators. *Phys. Rev. Lett.* **2012**, *109*, 097601.

(3) Baljozović, M.; Arnoldi, B.; Grass, S.; Lacour, J.; Aeschlimann, M.; Stadtmüller, B.; Ernst, K.-H. Spin- and Angle-Resolved Photoemission Spectroscopy Study of Heptahelicene Layers on Cu(111) Surfaces. *J. Chem. Phys.* **2023**, *159*, 044701.

(4) Kettner, M.; Maslyuk, V. V.; Nürenberg, D.; Seibel, J.; Gutierrez, R.; Cuniberti, G.; Ernst, K.-H.; Zacharias, H. Chirality-Dependent Electron Spin Filtering by Molecular Monolayers of Helicenes. *J. Phys. Chem. Lett.* **2018**, *9*, 2025–2030.

(5) Göhler, B.; Hamelbeck, V.; Markus, T. Z.; Kettner, M.; Hanne, G. F.; Vager, Z.; Naaman, R.; Zacharias, H. Spin Selectivity in Electron Transmission Through Self-Assembled Monolayers of Double-Stranded DNA. *Science* **2011**, *331*, 894–897.

(6) Duhm, S.; Gerlach, A.; Salzmann, I.; Bröker, B.; Johnson, R.; Schreiber, F.; Koch, N. PTCDA on Au (111), Ag (111) and Cu (111): Correlation of Interface Charge Transfer to Bonding Distance. *Org. Electron.* **2008**, *9*, 111–118.

(7) LaShell, S.; McDougall, B. A.; Jensen, E. Spin Splitting of an Au(111) Surface State Band Observed with Angle Resolved Photoelectron Spectroscopy. *Phys. Rev. Lett.* **1996**, *77*, 3419–3422.

(8) Petersen, L.; Hedegård, P. A Simple Tight-Binding Model of Spin–Orbit Splitting of *sp*-Derived Surface States. *Surf. Sci.* **2000**, *459*, 49–56.

(9) Hamann, D. R. Optimized Norm-Conserving Vanderbilt Pseudopotentials. *Phys. Rev. B* **2013**, *88*, 085117.

(10) Schlipf, M.; Gygi, F. Optimization Algorithm for the Generation of ONCV Pseudopotentials. *Comput. Phys. Commun* **2015**, *196*, 36–44.

(11) Berland, K.; Hyldgaard, P. Exchange Functional that Tests the Robustness of the Plasmon Description of the van der Waals Density Functional. *Phys. Rev. B* **2014**, *89*, 035412.

(12) Perdew, J. P.; Burke, K.; Ernzerhof, M. Generalized Gradient Approximation Made Simple. *Phys. Rev. Lett.* **1996**, *77*, 3865–3868.

(13) Giannozzi, P.; Andreussi, O.; Brumme, T.; Bunau, O.; Buongiorno Nardelli, M.; Calandra, M.; Car, R.; Cavazzoni, C.; Ceresoli, D.; Cococcioni, M. et al. Advanced Capabilities for Materials Modelling with QUANTUM ESPRESSO. *J. Phys.: Condens. Matter* **2017**, *29*, 465901.

(14) Liu, Z.-F.; da Jornada, F. H.; Louie, S. G.; Neaton, J. B. Accelerating GW-Based Energy Level Alignment Calculations for Molecule–Metal Interfaces Using a Substrate Screening Approach. *J. Chem. Theory Comput.* **2019**, *15*, 4218–4227.

(15) Deslippe, J.; Samsonidze, G.; Strubbe, D. A.; Jain, M.; Cohen, M. L.; Louie, S. G. BerkeleyGW: A Massively Parallel Computer Package for the Calculation of the Quasiparticle and Optical Properties of Materials and Nanostructures. *Comput. Phys. Commun.* **2012**, *183*, 1269–1289.

(16) Hybertsen, M. S.; Louie, S. G. Electron Correlation in Semiconductors and Insulators: Band Gaps and Quasiparticle Energies. *Phys. Rev. B* **1986**, *34*, 5390–5413.

(17) Ismail-Beigi, S. Truncation of Periodic Image Interactions for Confined Systems. *Phys. Rev. B* **2006**, *73*, 233103.

(18) Deslippe, J.; Samsonidze, G.; Jain, M.; Cohen, M. L.; Louie, S. G. Coulomb-Hole Summations and Energies for *GW* Calculations with Limited Number of Empty Orbitals: A Modified Static Remainder Approach. *Phys. Rev. B* **2013**, *87*, 165124.
